# Less Pain with Intra-Articular Hyaluronic Acid Injections for Knee Osteoarthritis Compared to Placebo: A Systematic Review and Meta-Analysis of Randomised Controlled Trials

**DOI:** 10.3390/ph17111557

**Published:** 2024-11-20

**Authors:** Filippo Migliorini, Nicola Maffulli, Luise Schäfer, Joshua Kubach, Marcel Betsch, Mario Pasurka

**Affiliations:** 1 Department of Orthopaedic and Trauma Surgery, Academic Hospital of Bolzano (SABES-ASDAA), 39100 Bolzano, Italy; luiseschaefer83@gmail.com; 2 Department of Life Sciences, Health, and Health Professions, Link Campus University, 00165 Rome, Italy; 3 Department of Trauma and Orthopaedic Surgery, Faculty of Medicine and Psychology, University La Sapienza, 00185 Roma, Italy; n.maffulli@qmul.ac.uk; 4 School of Pharmacy and Bioengineering, Faculty of Medicine, Keele University, Stoke on Trent ST4 7QB, UK; 5 Centre for Sports and Exercise Medicine, Barts and the London School of Medicine and Dentistry, Mile End Hospital, Queen Mary University of London, London E1 4DG, UK; 6 Department of Trauma Surgery and Orthopaedics, University Hospital Erlangen, Friedrich-Alexander-University Erlangen-Nuremberg, 90455 Erlangen, Germany; joshua.kubach@uk-erlangen.de (J.K.); marcel.betsch@uk-erlangen.de (M.B.); mario.pasurka@uk-erlangen.de (M.P.)

**Keywords:** knee, osteoarthritis, hyaluronic acid, infiltrations, placebo

## Abstract

The present meta-analysis investigated the efficacy of intra-articular hyaluronic acid (HA) injections for knee osteoarthritis. The outcomes of interest were the visual analogue scale (VAS) and Western Ontario McMaster Osteo-Arthritis Index (WOMAC) scores. This study was conducted according to the 2020 PRISMA statement. All the randomised controlled trials (RCTs) comparing the efficacy of intra-articular HA injections versus placebo injections for knee osteoarthritis were accessed in September 2024. Data from 3851 patients were collected. In total, 64% (2467 of 3851 patients) were women, and the mean age of the patients was 63.5 ± 4.9 years. At baseline, good comparability was found for the mean age, BMI, percentage of women, and patient-reported outcome measures (PROMs). Studies which reported data from two to four weeks of follow-up evidenced a lower value of the subscales pain (*p* < 0.0001) and stiffness (*p* = 0.01) of the WOMAC score. No difference was observed in VAS at rest (*p* = 0.4), VAS at exercise (*p* = 0.1), and subscale function (*p* = 0.4) of the WOMAC score. Studies which reported data from five to eight weeks of follow-up evidenced lower VAS at rest in favour of the HA group (*p* = 0.01). No difference in the other PROMs of interest was observed: VAS at exercise (*p* = 0.1), and the subscales pain (*p* = 0.3), function (*p* = 0.4), and stiffness (*p* = 0.4) of the WOMAC score. The current level I of evidence suggests that intra-articular HA injections in the knee might reduce pain in the short term.

## 1. Introduction

Osteoarthritis (OA) is a common joint ailment in adults worldwide, with the knee being the most affected joint (6% of all adults) [1,2,3,4,5]. The likelihood of developing knee OA increases with age and rises to 40% among patients between ages 70 and 74 [5,6,7,8,9,10]. Symptomatic knee OA leads to significant pain, reduction in mobility and independence, and decreases the patient’s quality of life [3,11,12,13,14,15]. Given the side effects of systemic non-steroidal anti-inflammatory drugs (NSAID) as the first-line treatment for symptomatic knee OA, intra-articular injections have been promoted [16]. In addition to corticosteroids and platelet-rich plasma, hyaluronic acid (HA) is the most commonly used agent for intra-articular treatments [16]. HA is a linear glycosaminoglycan produced at the plasma membrane by synthases. It possesses exceptional physicochemical properties, including biocompatibility, biodegradability, non-inflammatory behaviour, non-toxicity, and non-immunogenicity [17]. HA comprises repeating units of N-acetyl-D-glucosamine and D-glucuronic acid, with the monosaccharide units linked by alternating β-1,3 and β-1,4 glycosidic bonds [17,18]. As a physiological component of synovial fluid, HA has an average molecular weight (MW) of 6000 to 7000 kDa and is present in the knee at concentrations of 2 to 4 mg/mL [19]. Initially produced at high molar mass, HA undergoes degradation over time, which decreases its molecular weight [20]. A physiological component of the synovial fluid, HA has an average molecular weight (MW) of 6000 to 7000 kDa and is present in the knee at concentrations of 2 to 4 mg/mL [19]. HA is produced at high molar mass, but, given its degradation, its mass progressively decreases [20]. In osteoarthritic knees, HA synthesis, degradation, and clearance are abnormal [21]. This leads to a reduced MW and HA concentration in the joint, inducing changes in synovial fluid viscoelasticity and subsequent cartilage damage [21]. The human body continuously requires newly produced high MW HA, making it essential to stimulate HA production. Honey can stimulate HA production in the skin [22]. Combining honey with HA can enhance wound healing through synergistic effects [23]. Given its viscoelastic, chondroprotective, and anti-inflammatory effects and its contribution to proteoglycan synthesis and scaffolding, intra-articular injections of HA may help restore articular homoeostasis [21,24]. Many HA preparations are currently available; they differ in their MW (500 kDa to >6000 kDa), production method, and structure [21,25] and have different rheological properties [26]. Although intra-articular visco-supplementation with HA has been extensive [27], there are inconsistent results [26], and its comparative efficacy remains controversial [28].

The present meta-analysis investigated the current level 1 evidence regarding the efficacy of intra-articular HA infiltrations for knee OA and their effects on patient outcomes measured by patient-reported outcome measures (PROMs). The objectives were to establish whether intra-articular HA infiltrations are associated with greater visual analogue scale (VAS) and Western Ontario McMaster Osteo-Arthritis Index (WOMAC) scores compared to placebo injections.

## 2. Methods

### 2.1. Eligibility Criteria

All randomised controlled trials (RCTs) comparing the efficacy of intra-articular HA infiltrations versus placebo injections for knee OA were accessed. Articles in English, German, Italian, French and Spanish articles were eligible. Additionally, only studies clearly stated that the infiltrations conducted in the knee were eligible. Studies which compared HA with other biologically active non-HA treatments (e.g., platelet-rich plasma, corticosteroids, mesenchymal stem cells) were not included. Only studies with evidence level I were considered [29]. Studies which evaluated intra-articular HA infiltrations augmented with other biologically active compounds were also not considered. Studies that were regarded as comparators of other non-injection therapies were not eligible.

### 2.2. Search Strategy

The 2020 Preferred Reporting Items for Systematic Reviews and Meta-Analyses (PRISMA) were followed [30]. The PICOTD algorithm was preliminarily established:

- P (Problem): OA of the knee;
- I (Intervention): intra-articular HA infiltrations;
- C (Comparison): placebo infiltrations;
- O (Outcomes): PROMs.
- T (Timing): two to eight weeks follow-up;
- D (Design): RCT.

PubMed, Web of Science, and Embase were accessed in September 2024. No time constraint was set for the search. The Medical Subject Headings (MeSH) used for the database search were: ((((“Osteoarthritis, Knee”[Mesh] OR knee osteoarthritis OR knee OA) AND (“Hyaluronic Acid”[Mesh] OR hyaluronic acid infiltrations OR HA infiltrations)) AND (“Placebos”[Mesh] OR Placebo)) AND (“Patient Outcome Assessment”[Mesh] OR “Patient Reported Outcome Measures”[Mesh] OR PROM OR visual analogue scale OR VAS OR Western Ontario and McMaster Universities Osteoarthritis OR WOMAC)) NOT (Hip). No additional filters were used in the database search.

### 2.3. Selection and Data Collection

Two authors (**and**) independently performed selection and data collection. The resulting titles were examined by hand. If accessible, the full text of the abstracts of interest was accessed. The bibliography of the full-text articles was also examined for potential inclusion. A senior author (**) made the final decision in case of reviewer disagreements.

### 2.4. Data Items

Data extraction was performed by two authors (**and**) independently in Microsoft Office Excel version 16.72 (Microsoft Corporation, Redmond, WA, USA). Author, year of publication and journal, length of the follow-up, and number of patients with related mean age and BMI were extracted. Data concerning the following PROMs were collected at baseline and the last follow-up: VAS at rest and during exercise [31], overall WOMAC and related subscales of pain, stiffness, and function [32]. Results from each RCT were grouped according to the following follow-ups: two to four weeks and five to eight weeks. The endpoint of interest was to investigate whether intra-articular HA infiltrations are associated with improved VAS and WOMAC scores compared to placebo injections at different follow-up times (two to four and five to eight weeks).

### 2.5. Methodological Quality Assessment and Quality of the Recommendations

The risk of bias was evaluated independently following the guidelines in the Cochrane Handbook for Systematic Reviews of Interventions Studies [33]. Disagreements were solved by a senior author (**). RCTs were assessed using the revised Risk of Bias assessment tool (RoB2) [34,35] of the Cochrane tool (The Nordic Cochrane Collaboration, Copenhagen, Denmark). The bias arising from the randomisation process, bias based on the deviations from intended interventions, bias of missing outcome data, bias in the measurement of the outcome, and bias in the selection of the reported result were evaluated.

### 2.6. Synthesis Methods

The main author (**) performed the statistical analyses. The guidelines of the Cochrane Handbook for Systematic Reviews of Interventions [36]. The IBM SPSS software version 25 (International Business Machines Corporation, Armonk, NY, USA) was used for descriptive statistics. The arithmetic mean and standard deviation were used. The meta-analyses were conducted using the Review Manager software version 5.3 (The Nordic Cochrane Collaboration, Copenhagen, Denmark). The inverse variance method with mean difference (MD) effect measure was used for continuous data. The Mantel–Haenszel method with odd ratio (OR) effect measure was used for binary data. The confidence interval (CI) was 95% in all the comparisons. Heterogeneity was evaluated through Higgins-I^2^ and χ^2^ tests. If P_χ2_ > 0.05, no statistically significant heterogeneity was found. If P_χ2_ < 0.05, the heterogeneity was assessed following the values of the Higgins-I^2^. Suppose the Higgins-I^2^ test > 50% high heterogeneity was found. A fixed effect model was set as default. If high heterogeneity was detected, a random model effect was used. Overall values of *p* < 0.05 were considered statistically significant.

## 3. Results

### 3.1. Study Selection

**Figure 1:**
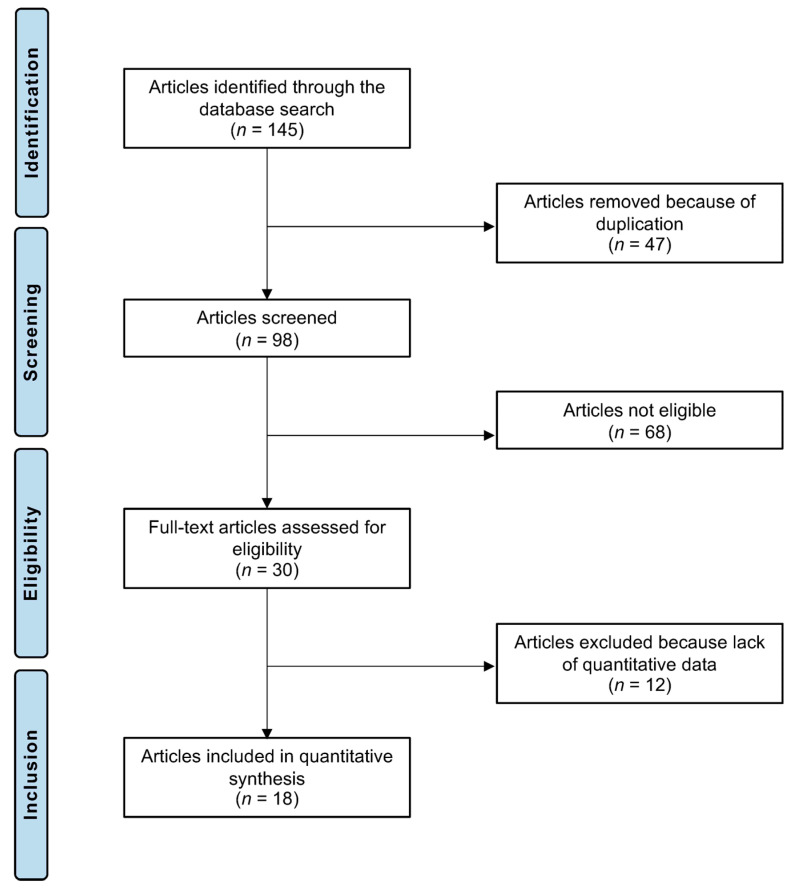
PRISMA flow chart of the literature search.

The systematic literature search resulted in 145 articles. Of them, 47 were excluded as they were duplicates. The remaining 98 investigations were screened for eligibility by reviewing the abstracts. A further 68 articles were discarded as they did not match the predefined eligibility criteria for the following reasons: study type and design (*n* = 16), low level of evidence (*n* = 9), not clearly stating that the infiltrations were conducted in the knee (*n* = 5), considering other non-infiltrative therapies as comparators (*n* = 11), comparing HA with other biologically active non-HA treatments (*n* = 12), evaluating intra-articular HA infiltrations augmented with other biologically active compounds (*n* = 9), and language limitations (*n* = 6). An additional 12 studies were not considered as they missed quantitative data under the outcomes of interest. Finally, 18 RCTs were selected for inclusion in the present investigation. The results of the literature search are shown in Figure 1.

### 3.2. Methodological Quality Assessment

**Figure 2:**
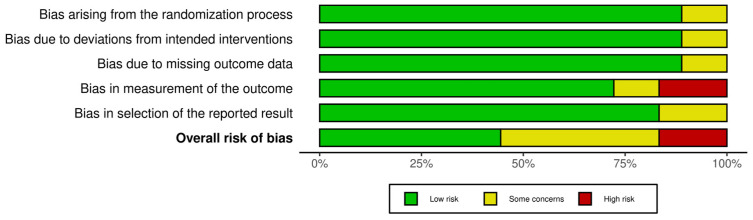
Cochrane risk of bias tool (RoB2).

To evaluate the risk of bias in the randomised controlled trials (RCTs) included in this meta-analysis, we employed the revised Risk of Bias assessment tool (RoB2). Most studies demonstrated high-quality allocation concealment, yielding comparable baseline groups and contributing to a low risk of bias in the randomisation process. However, some studies showed concerns regarding deviations from the intended intervention, missing outcome data, and selective outcome reporting, resulting in a low to moderate risk of bias in these areas. Additionally, due to the lack of blinding of outcome assessors to intervention status, a high risk of bias was identified in three studies when measuring outcomes. In summary, the risk of bias analysis, as illustrated in Figure 2, reflects an overall low to moderate quality in the methodological rigour of the included RCTs.

### 3.3. Study Characteristics and Results of Individual Studies

Data from 3851 patients were collected. In total, 64% (2467 of 3851) of patients were women, and the mean age of the patients was 63.5 ± 4.9 years. The generalities and demographics of the included studies are shown in Table 1.

### 3.4. The Baseline of the Groups

At baseline, the mean age, BMI, percentage of women, and PROMs were comparable (Table 2).

### 3.5. Meta-Analyses

**Figure 3:**
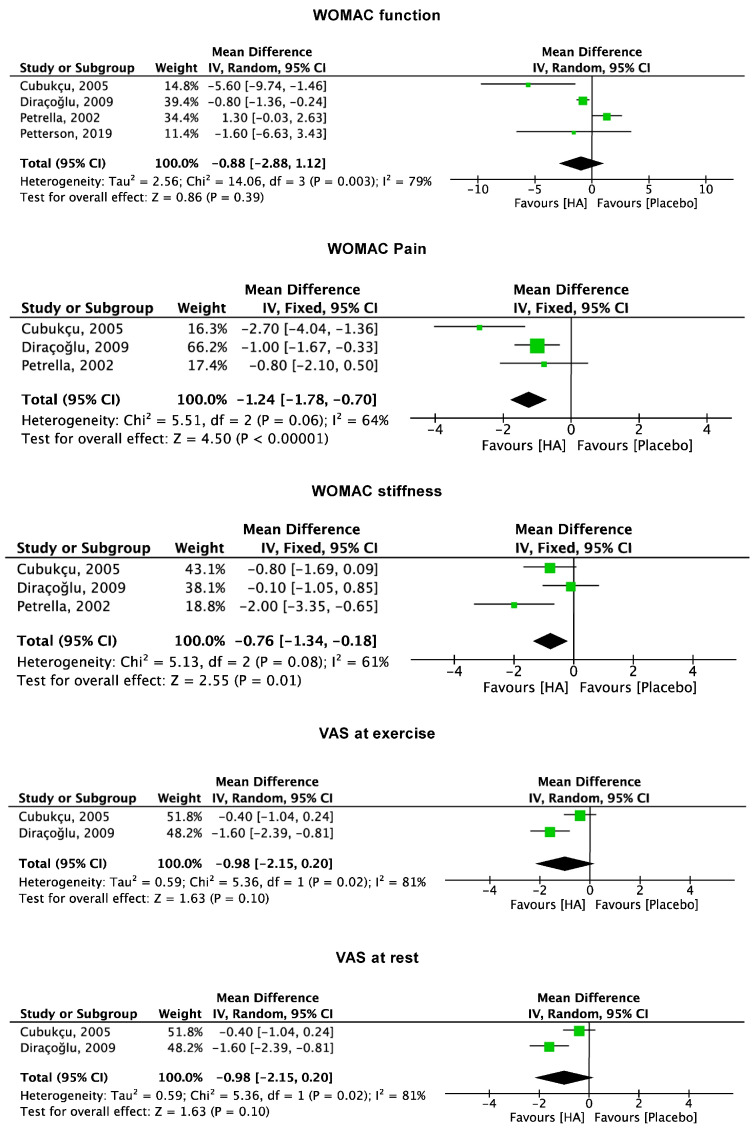
Results of the studies reporting data on VAS and WOMAC at two to four weeks follow-up. (VAS: visual analogue scale; WOMAC: Western Ontario and McMaster Universities Osteoarthritis; CI: confidence interval).

Seven studies [39,40,42,45,47,51,52] compared HA vs. placebo and reported finite values of VAS and WOMAC, which were included in the meta-analyses. Studies which reported data from two to four weeks [40,42,51,52] of follow-up evidenced a lower value of the subscales pain (MD −1.24; 95% CI −1.78 to −0.70; *p* < 0.0001) and stiffness (MD −0.76; 95% CI −1.34 to −0.18; *p* = 0.01) of the WOMAC score. No difference was observed in VAS at rest (*p* = 0.4), VAS at exercise (*p* = 0.1), and subscale function (*p* = 0.4) of the WOMAC score (Figure 3).

**Figure 4:**
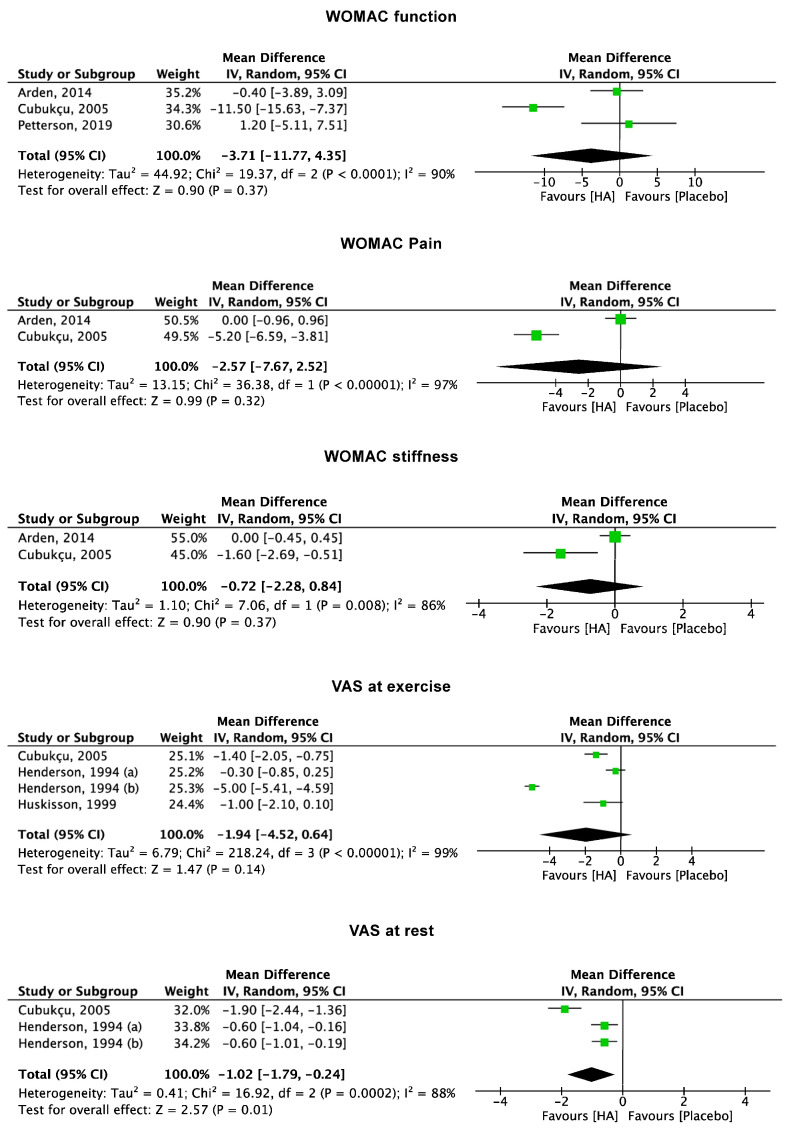
Results of the studies reporting data on VAS and WOMAC at five to eight weeks follow-up. (VAS: visual analogue scale; WOMAC: Western Ontario and McMaster Universities Osteoarthritis; CI: confidence interval).

Studies reporting data from five to eight weeks [39,40,45,47,52] of follow-up evidenced lower VAS at rest in favour of the HA group (MD −1.02; 95% CI −1.79 to 0.24; *p* = 0.01). No difference was observed in the other PROMs of interest: VAS at exercise (*p* = 0.1) and the subscales pain (*p* = 0.3), function (*p* = 0.4), and stiffness (*p* = 0.4) of the WOMAC score (Figure 4).

## 4. Discussion

According to the main findings of the present meta-analysis, the currently available level I of evidence suggests that intra-articular HA injections promote a short-term reduction in pain in patients with knee OA. Studies which reported data from two to four weeks of follow-up evidenced a lower value of the WOMAC subscales pain and stiffness. No differences were observed in VAS at rest, VAS at exercise, and the WOMAC subscale function. Studies which reported data from five to eight weeks of follow-up evidenced lower VAS at rest in favour of the HA group. However, no differences in VAS at exercise and the WOMAC subscales were observed in pain, function and stiffness.

HA injection therapy is well tolerated, with only limited local discomfort and the absence of systemic side effects [24]. HA contributes to normal articular homoeostasis as a physiologic component of the synovial fluid [24]. With its chondroprotective and anti-inflammatory effects and contribution to proteoglycan synthesis and scaffolding, previously published studies suggested that intraarticular injections of HA may improve pain and function in OA patients in the short- [40,41,42,52], mid- [27,37,38,41] and long-term [43] compared to placebo injections. However, there is still no consensus about the effectiveness of intra-articular treatment with HA because of contrasting outcomes in different clinical studies. Given the lack of evidence, the present level I systematic review and meta-analysis was conducted.

Previously published studies agree with the findings of significant short-term OA knee pain relief and improvements in knee joint function after HA injection compared to a placebo [37,40,41,42]. Repeated injections may be beneficial as HA degrades and molecular mass decreases [20]. However, this was not the scope of the present analysis. While most of the included trials followed a protocol of one weekly injection for three weeks [38,40,41,42], one HA injection is also safe and effective, providing a clinically meaningful reduction in knee pain [37,52]. Comparing single injection with multi-injection (3–5 injections) regimens, a meta-analysis reported similar results regarding relief in patients with knee OA [55]. Altman et al. investigated the effect of a single injection of non-animal stabilised hyaluronic acid (NASHA) compared with placebo in patients with knee OA [37]. Interestingly, WOMAC scores and quality of life were improved in both groups, with no statistically significant between-group differences in response rates for any efficacy parameters for the overall population (generalised OA and knee OA) [37]. However, in their subgroup analysis, patients with knee OA demonstrated a greater response following NASHA treatment compared with the overall study population [37]. The maximum response rate of 36.6% occurred at six weeks post-treatment [37]. In line with these findings, in a multicenter, double-blinded, randomised, placebo-controlled trial, the HA group experienced a significantly greater improvement in the WOMAC pain score through week 26 than the placebo [52]. In contrast, a multicenter double-blinded randomised placebo-controlled trial with a minimum 26-week follow-up showed a strong placebo effect and could not show the superiority of a single HA injection over a placebo injection [49]. Significant reductions in WOMAC A1 scores were observed in both treatment groups compared to baseline at 26 weeks post-injection [49]. These conflicting results may be caused by different HA products with varying concentrations and volumes.

Studies following a protocol of multiple injections reported a significant reduction in pain and function scores, particularly in short-term follow-ups. In a prospective, randomised, placebo-controlled clinical trial, pain at rest decreased from the 3rd week to the 8th week after injection [40]. Night pain, pain during walking, and the need for paracetamol in the HA group were significantly lower than in the saline group at the 8th-week follow-up [40]. Also, WOMAC pain score reduction began in the 3rd week after injection and continued until week 8 [40]. However, in the present meta-analysis, significant differences in the WOMAC subscale pain were only found at the two to four-week follow-up rather than at the five to eight-week follow-up. Longer follow-ups with multiple HA injections show inconsistent results. Three weekly injections of a high MW HA derived from nonpyogenic streptococcus zooepidemicus resulted in significant knee OA pain relief at 26 weeks compared with a control group with an intraarticular injection of buffered saline [38]. By the end of the treatment series, both groups demonstrated a reduction in pain scores. However, the control group demonstrated a lessening effect over time, with significant differences in VAS at 26 weeks [38]. Significant differences in the reduction in WOMAC scores were only obtained in some subscales [38]. Mild to moderate knee OA patients experienced improvements in WOMAC function scores, with significantly greater pain reduction compared to age and diseased-matched control patients receiving intra-articular placebo [41]. However, six months post-treatment, the relative and absolute improvements in the pain scores of the HA group did not meet the OMERACT-OARSI set of intervention responder criteria [41,56]. Some factors may influence the treatment response of HA injection therapy. In addition to the severity of radiographic OA changes [45,57,58], cultural and environmental factors could play a role [49]. Additionally, Chinese versus European patients observed a higher overshadowing placebo effect [49]. Besides different injection protocols, the studies also used different HA MW. To date, the influence of MW on treatment efficacy is unclear, and there is no consensus on which MW in HA products is the best option for knee OA [59]. Higher molecular weight HA injections have been suggested to be more effective, possibly because they more closely reflect the HA in the knee joint, which contains HA of MW between 2000 and 10,000 kD [60,61,62,63,64]. However, a network meta-analysis of randomised controlled trials showed mixed results when comparing HA with different molecular weights [59]. In addition to our findings on significant short-term effects of HA, there is also some evidence that HA might lead to long-term beneficial effects. A one-year placebo-controlled trial showed that, in addition to short-term improvements in pain and function scores, the need to perform supplementary local therapies was more frequent in the placebo group at 1-year follow-up [43]. Even after one year, the improvement in the functional index was statistically significantly greater in the HA group [43].

Despite showing short-term improvements in pain and function scores following HA injections compared to placebo, there is also some evidence that the difference in efficacy is not that large [28]. In a systematic review and meta-analysis of randomised controlled trials, there was no significant difference in pain reduction in the HA groups compared to the placebo groups at the 3-month follow-up [28]. There may also be adverse effects after HA injection, such as a transient increase in pain and swelling in the affected knee [45], suggesting that HA or one of its metabolites may act as an irritant or inflammatory mediator in some patients. However, these adverse effects usually lasted less than four days [45].

The severity of knee OA plays a crucial role in the efficacy of HA treatment. HA injections can improve joint function and reduce symptoms in mild to moderate OA by restoring synovial fluid properties. However, in advanced OA, where cartilage degradation is significant, HA’s effectiveness may decrease given the limited improvement in joint lubrication and cartilage protection. This variability in OA severity across studies can lead to heterogeneous results, affecting the generalizability of conclusions. Future studies should stratify participants by OA severity to better understand how treatment responses vary across disease stages and provide more targeted recommendations.

This study has some limitations. First, the severity of knee OA was not homogeneous among studies. Previous studies showed that patients with severe radiographic changes (Kellgren–Lawrence 4) are less responsive to HA therapy. In contrast, patients with low to moderate radiographic changes (Kellgren–Lawrence 2–3) seem to respond better [45,57,58]. A subgroup analysis may have given us more accurate information. However, this was not possible because of missing data. Second, the included studies followed a different infiltration protocol. While some studies investigated the effects of a single intraarticular injection of HA or placebo [27,37,49,52], others applied multiple injections [38,40,41,46,65].

The included studies were published between 1994 and 2021, and most included clinical trials are not recent. The lack of recent clinical studies on the efficacy of HA, particularly for knee OA, may arise from updated guidelines, a shift towards exploring alternative treatments like platelet-rich plasma (PRP) or mesenchymal stem cells [66,67,68,69], and existing evidence suggesting only modest benefits. Consequently, recent research focused on meta-analyses and reviews rather than generating new trials, with greater attention directed toward therapies with more robust or emerging support. In addition to varying injection protocols, MW, which directly influences the rheological properties of the respective HA preparation [26], differed between studies. The heterogeneity of HA products necessitates future studies to compare the different HA products and determine which molecular weight range and injection protocol is the best option for knee OA. The polydispersity index (PDI) was not included in the present analysis, as most studies did not report it. Therefore, the influence of PDI cannot be determined with this analysis. The patient populations of included studies were rather old. Younger populations which could develop joint issues may also benefit from a temporary treatment with HA. This would be an interesting area for future research.

## 5. Conclusions

Current level I evidence suggests that intra-articular HA injections may reduce pain in the short term. However, they do not affect function, with response varying by duration after injection. The severity of OA differs across studies and may influence treatment efficacy. Future research should compare HA products with different molecular weight ranges and injection protocols.

## Figures and Tables

**Table 1:** Generalities and demographics of the included studies (HA: hyaluronic acid).

Author, Year	Journal Name	Intervention	Patients (*n*)	Mean Age (*y*)	Women (%)
Altman et al., 2004 [37]	*Osteoarthritis Cartilage*	HA	173	62.9	46
		Placebo	174	63.3	64
Altman et al., 2009 [38]	*Semin Arthritis Rheum*	HA	293	62.5	63
		Placebo	295	60.8	63
Arden et al., 2014 [39]	*Curr Med Res Opin*	HA	108	64.5	55
		Placebo	110	60.9	46
Cubukçu et al., 2005 [40]	*Clin Rheumatol*	HA	20	52.6	70
		Placebo	10	57.6	10
DeCaria et al., 2011 [41]	*Arch Gerontol Geriatr*	HA	15	71.9	47
		Placebo	15	72.9	47
Diraçoğlu et al., 2009 [42]	*J Back Musculoskelet Rehabil*	HA	42	59.4	90
		Placebo	21	56.2	100
Dougados et al., 1993 [43]	*Osteoarthritis Cartilage*	HA	55	67.0	78
		Placebo	55	69.0	65
Hangody et al., 2018 [44]	*Cartilage*	HA	149	57.5	65
		Placebo	69	58.0	74
		HA	150	59.2	66
		Placebo	69	58.0	74
Henderson et al., 1994 [45]	*Ann Rheum Dis*	HA	10	63.9	50
		Placebo	26	67.0	69
		HA	25	72.1	80
		Placebo	20	60.0	75
Henderson et al., 1994 [45]	*Ann Rheum Dis*	HA	10	63.9	50
		Placebo	26	67.0	69
		Placebo	20	60.0	75
		HA	25	72.1	80
Huang et al., 2011 [46]	*BMC Musculoskelet Disord*	HA	100	65.9	74
		Placebo	100	64.2	78
Huskisson et al., 1999 [47]	*Rheumatology (Oxford)*	HA	50	65.8	76
		Placebo	50	64.8	58
Karlsson et al., 2002 [48]	*Rheumatology (Oxford)*	HA	92	72.0	67
		Placebo	66	71.0	61
		HA	88	70.0	65
		Placebo	66	71.0	61
Ke et al., 2021 [49]	*BMC musculoskeletal disorders*	HA	218	61.5	77
		Placebo	220	61.6	78
Lin et al., 2019 [50]	*Arthroscopy*	HA	27	62.5	66
		Placebo	29	62.2	63
Petrella et al., 2002 [51]	*Arch Intern Med*	HA	30	67.3	36
		Placebo	30	62.6	43
Petterson et al., 2019 [52]	*Knee Surg Sports Traumatol Arthrosc*	HA	184	59.5	59
		Placebo	185	58.7	57
Pham et al., 2004 [53]	*Ann Rheum Dis*	HA	131	64.9	71
		Placebo	85	64.9	61
van der Weegen et al., 2014 [54]	*J Arthroplasty*	HA	99	58.7	51
		Placebo	97	60.1	48

**Table 2:** Baseline comparability. (BMI: body mass index; VAS: visual analogue scale; WOMAC: Western Ontario and McMaster Universities Osteoarthritis).

Endpoints	P
Mean age (*y*)	0.1
Women (%)	0.8
Mean BMI (kg/m^2^)	0.1
VAS at rest	0.4
VAS at exercise	0.4
WOMAC total	0.2
WOMAC pain	0.9
WOMAC stiffness	0.5
WOMAC function	0.3

## Data Availability

The datasets generated during and/or analysed during the current study are available throughout the manuscript.
